# Hemodynamic coherence and the rationale for monitoring the microcirculation

**DOI:** 10.1186/cc14726

**Published:** 2015-12-18

**Authors:** Can Ince

**Affiliations:** 1 Department of Intensive Care, Erasmus MC University Hospital Rotterdam, P.O. Box 2040, 3000 CA, Rotterdam, the Netherlands

## Abstract

This article presents a personal viewpoint of the shortcoming of conventional hemodynamic resuscitation procedures in achieving organ perfusion and tissue oxygenation following conditions of shock and cardiovascular compromise, and why it is important to monitor the microcirculation in such conditions. The article emphasizes that if resuscitation procedures are based on the correction of systemic variables, there must be coherence between the macrocirculation and microcirculation if systemic hemodynamic-driven resuscitation procedures are to be effective in correcting organ perfusion and oxygenation. However, in conditions of inflammation and infection, which often accompany states of shock, vascular regulation and compensatory mechanisms needed to sustain hemodynamic coherence are lost, and the regional circulation and microcirculation remain in shock. We identify four types of microcirculatory alterations underlying the loss of hemodynamic coherence: type 1, heterogeneous microcirculatory flow; type 2, reduced capillary density induced by hemodilution and anemia; type 3, microcirculatory flow reduction caused by vasoconstriction or tamponade; and type 4, tissue edema. These microcirculatory alterations can be observed at the bedside using direct visualization of the sublingual microcirculation with hand-held vital microscopes. Each of these alterations results in oxygen delivery limitation to the tissue cells despite the presence of normalized systemic hemodynamic variables. Based on these concepts, we propose how to optimize the volume of fluid to maximize the oxygen-carrying capacity of the microcirculation to transport oxygen to the tissues.

## Introduction

Resuscitating critically ill patients from states of shock and cardiovascular compromise remains a challenge in intensive care medicine. Procedures currently in place in guidelines mainly focus on the administration of fluids and on vasoactive medications targeting the normalization of systemic hemodynamic parameters such as cardiac output, blood pressure variables, and venous saturation. In support of this approach, the blinded, randomized controlled trial (RCT) with large numbers of patients has been propagated as the method to provide evidence for the clinical benefit of targeting various systemic variables. Such an approach precludes a mechanistic approach, which is explicitly excluded from the RCT design. However, studies in critically ill patients comparing various interventions have shown either no or marginal differences between groups (e.g., [1-7]). A major problem in the interpretation of these trials is that no distinction can be made between whether there is indeed no difference between the different interventions or whether the trial design has been ineffective in demonstrating a difference. The latter is a likely possibility due to the heterogeneity of the patient population and lack of uniform and standardized protocolized treatments. Unfortunately, the conclusions often drawn are that there is no difference between the investigated procedures or that the procedures are ineffective. To make a distinction between two different interventions, a more physiological approach that emphasizes whether the investigated procedures were effective in accomplishing the mechanistic expectation is needed. For example, in the context of fluid resuscitation, it is important to establish whether the resuscitation procedure under investigation effectively improves tissue perfusion and oxygenation because it is this improvement which must be considered as the ultimate purpose of resuscitation [8].

Shock in need of resuscitation is a condition in which oxygen delivery to the cells is insufficient to sustain cellular activity and support of organ function [9]. Shock in this context is defined at a cellular level. Resuscitation targets, if at all applied, are conventionally aimed at the normalization of systemic variables of circulation and oxygenation because it is expected that normalization of such systemic variables will result in a parallel improvement in the perfusion of the microcirculation and will achieve restoration of tissue oxygenation. Hemodynamic coherence is what we have termed the condition in which this parallel improvement is in place. However, in many conditions of resuscitation following an episode of shock, there is a loss of this coherence. The microcirculation and its tissues can remain hypoperfused despite the correction of systemic variables by fluids and vasoactive compounds.

## What is hemodynamic coherence?

Hemodynamic coherence between the macrocirculation and the microcirculation is the condition in which resuscitation procedures aimed at the correction of systemic hemodynamics variables are effective in correcting regional and microcirculatory perfusion and oxygen delivery to the parenchymal cells such that the cells are able to perform their functional activities in support of organ function. Many studies in the literature have described conditions of a loss of hemodynamic coherence where resuscitation resulted in a normalization of systemic hemodynamic variables but did not lead to a parallel improvement in microcirculatory perfusion and oxygenation [10-14]. The concept of a loss of hemodynamic coherence probably explains the negative results in the 1990s where several studies were conducted that targeted the normalization, or even the supranormalization, of systemic oxygen delivery variables [15,16]. Indeed, if there had been a loss of hemodynamic coherence in these patients, which is likely, then normalizing the systemic circulation without a parallel improvement in the peripheral and microcirculation of the various organ beds would indeed have been futile

For hemodynamic coherence to be effective, resuscitation based on the administration of fluids and blood, in combination with administered vasoactive compounds, must result in the effective delivery of oxygen-carrying blood in proportion to the various organ beds in a manner that matches oxygen supply to the heterogeneous oxygen demand of the various organs and their parenchymal cells. For hemodynamic coherence to be effective, the compensatory mechanisms, including hormonal, neural, and biochemical and vascular regulatory control systems, must be intact and able to sense and regulate oxygen transport to the various tissues. However, states of shock, reperfusion, inflammation, and infections can damage the cellular sensing mechanisms needed to regulate blood flow. In these cases, simply restoring systemic hemodynamic abnormalities becomes ineffective in restoring the microcirculation and in correcting tissue hypoperfusion. The pathogenic mechanisms underlying the loss of hemodynamic coherence include the generation of reactive nitrosative and oxidative species, which results in the loss of vascular regulation, in compromised endothelial cell function, and in compromised barrier function resulting in tissue hypoxemia. In addition, the resuscitation procedures themselves can actually interfere with the ability of the cardiovascular system to ensure effective distribution of oxygenated blood to the various organ beds. For example, fluid resuscitation due to hemodilution reduces blood viscosity, an essential physiological hemorheological variable needed for sheer stress-mediated vasotone regulation [17]. Fluids can also induce oxidative and nitrosative stress as well reduce the oxygen-carrying capacity of blood by hemodilution, both of which can contribute to reduced vascular regulatory capacity, loss of hemodynamic coherence, and reduced oxygen-carrying capacity to vulnerable organs such as the kidney (e.g., [18]). In addition vasoactive medications such as vasopressors and dilators can overwhelm endogenous receptor-mediated vasoregulation, further contributing to loss of hemodynamic coherence.

Metabolic demands and the matching of oxygen supply to demand heterogeneity occur not only between organs but also within organs, between different cells, and even at the subcellular level where there is a heterogeneity of oxygen consumption between mitochondria [19]. It is obvious that the regulation of blood flow and oxygen transport is a highly complex and regulated system that integrates cellular needs with vascular regulation mechanisms. Thus, for hemodynamic monitoring to be successful in guiding therapy, the monitoring of systemic parameters such as blood pressure and cardiac output need to be expanded by monitoring of the microcirculation [20].

States of shock can be accompanied by an intact hemodynamic coherence or a loss of hemodynamic coherence. The condition of preserved hemodynamic coherence between the macrocirculation and the microcirculation of different organ systems, for example, was shown in a study by van Iterson et al. using a hemorrhagic shock and blood resuscitation model in pigs. In the study, some animals responded to resuscitation while others did not. However, in both groups there was coherence between the behavior of the systemic hemodynamic variables and the regionally measured intestinal and heart microvascular oxygen pressures [21]. In a cardiac tamponade model of obstructive shock in pigs, fluid resuscitation was applied to normalize cardiac output to baseline levels. Van Genderen et al. reported that coherence was found in the response of the regional microcirculatory perfusion of the gut, muscle, and sublingual microcirculation, thereby demonstrating coherence between the macrocirculation and the microcirculation. In contrast, they found that fluid resuscitation applied following endotoxemic shock was not successful in restoring microcirculatory perfusion to baseline despite normalization of cardiac output and mean arterial pressure [22]. Several other clinically relevant large animal studies in sepsis have investigated the presence of hemodynamic coherence between the microcirculation of different organ systems. In a hyperdynamic model of cholangitis in pigs, Verdant et al. [23] showed coherence between microcirculatory alterations in the gut and the sublingual microcirculation. Dubin et al., on the contrary, found in sheep in endotoxic shock that there was hemodynamic coherence between the different systemic and microcirculatory compartments where blood pressure, cardiac output, mesenteric blood flow, as well as sublingual and gut serosal and mucosal microcirculation were similarly affected. Lack of coherence appeared during the resuscitation phase, however, where systemic and intestinal hemodynamics and sublingual and serosal microcirculation were restored but the intestinal villi remained hypoperfused [24].

The loss of coherence between different compartments in a single organ system can also occur, which was observed in a pig endotoxemia model by Siegemund et al. The authors found that fluids were effective in restoring mucosal microcirculatory oxygenation but were ineffective in restoring the oxygenation of the intestinal serosa [25]. Immunohistochemistry demonstrated that this lack of coherence was caused by the heterogeneous distribution of endotoxin-induced, inducible nitric oxide synthase enzyme between the mucosa and serosa of the intestines, and this caused abnormal flow regulation in the intestines [26].

Clinical studies monitoring sublingual microcirculation using hand-held videomicroscopy have identified the lack of hemodynamic coherence in several studies, a condition associated with increased morbidity and mortality [10-13]. An early study by LeDoux et al. [27] used laser Doppler measurements and other surrogates of microcirculatory perfusion in septic shock patients, and they found that while norepinephrine was successful in increasing blood pressure, it was ineffective in recruiting the microcirculation. A multicenter survey in three countries found no relationship between altered sublingual microcirculation and systemic variables, but there was a strong correlation between microcirculatory alterations, lactate levels, and the use of vasopressor therapy [28]. In the perioperative phase, the loss of coherence between mean arterial pressure and the microcirculation was found in cardiopulmonary bypass patients in whom arterial pressure was controlled using inotrope/vasopressor agents [29]. In other studies, however, the therapeutic targeting of stroke volume, but not of venous pressure, resulted in coherence between the systemic and the microcirculations [30]. den Uil et al. [31] reported that some patients in cardiogenic shock being treated with various inotropic agents experienced a lack of normalization of the microcirculation despite treatment and normalization of the cardiac index, and they found this condition to be associated with increased mortality. In traumatic hemorrhagic shock patients, Tachon et al. were successful in restoring systemic hemodynamic parameters by applying fluids, blood, and vasoactive medication in the resuscitation phase. However, despite the almost immediate correction of cardiac output and arterial pressure, the sublingual microcirculation took up to 4 days to recover, with the length of time to recover correlating with the occurrence of organ dysfunction [13].

Loss of hemodynamic coherence is most frequently found in septic patients in whom a lack of microcirculatory recruitment is observed despite successful macrocirculatory resuscitation (e.g., [11,12,32]). If there are also macrocirculatory abnormalities in addition to microcirculatory alterations, this can be a sign of extra risk for adverse outcomes. This possibility was recently shown in a multicenter, international microcirculatory observational study carried out by Vellinga et al. in 501 intensive care patients from 36 ICUs worldwide. The authors found that a high heart rate was an independent risk factor for in-hospital mortality, and also that if such a systemic abnormality was associated with microcirculatory alterations then the chance of in-hospital mortality almost doubled [33].

Clinical studies also have looked at the coherence between the microcirculation of different organ systems. These studies have predominantly studied the relationship between sublingual and intestinal microcirculation measured in the ileostoma of patients. Boerma et al. identified in such a study that time is an important parameter in the establishment of coherences. In the early stages of sepsis, there was no coherence between the intestinal and sublingual microcirculation, but after 3 days, when the septic insult generalized, there was coherence between the sublingual and intestinal microcirculation [34]. Edul et al. reported a heterogeneous response to a fluid challenge between the macrocirculation and intestinal and sublingual microcirculations in septic patients with ileostomies. They found that while macrocirculatory parameters increased without exception (cardiac index, mean arterial pressure, central venous pressure, and abdominal pressure) to a fluid challenge, neither sublingual nor intestinal microcirculation responded in parallel, and there was no coherence between the sublingual and intestinal microcirculation changes. The sublingual microcirculation was in coherence with increases in cardiac output and dependent on the initial state of the microcirculation, but the intestinal microcirculation remained completely unresponsive to fluid challenge [35].

## Four types of microcirculation alterations underlying the loss of hemodynamic coherence

**Figure 1:**
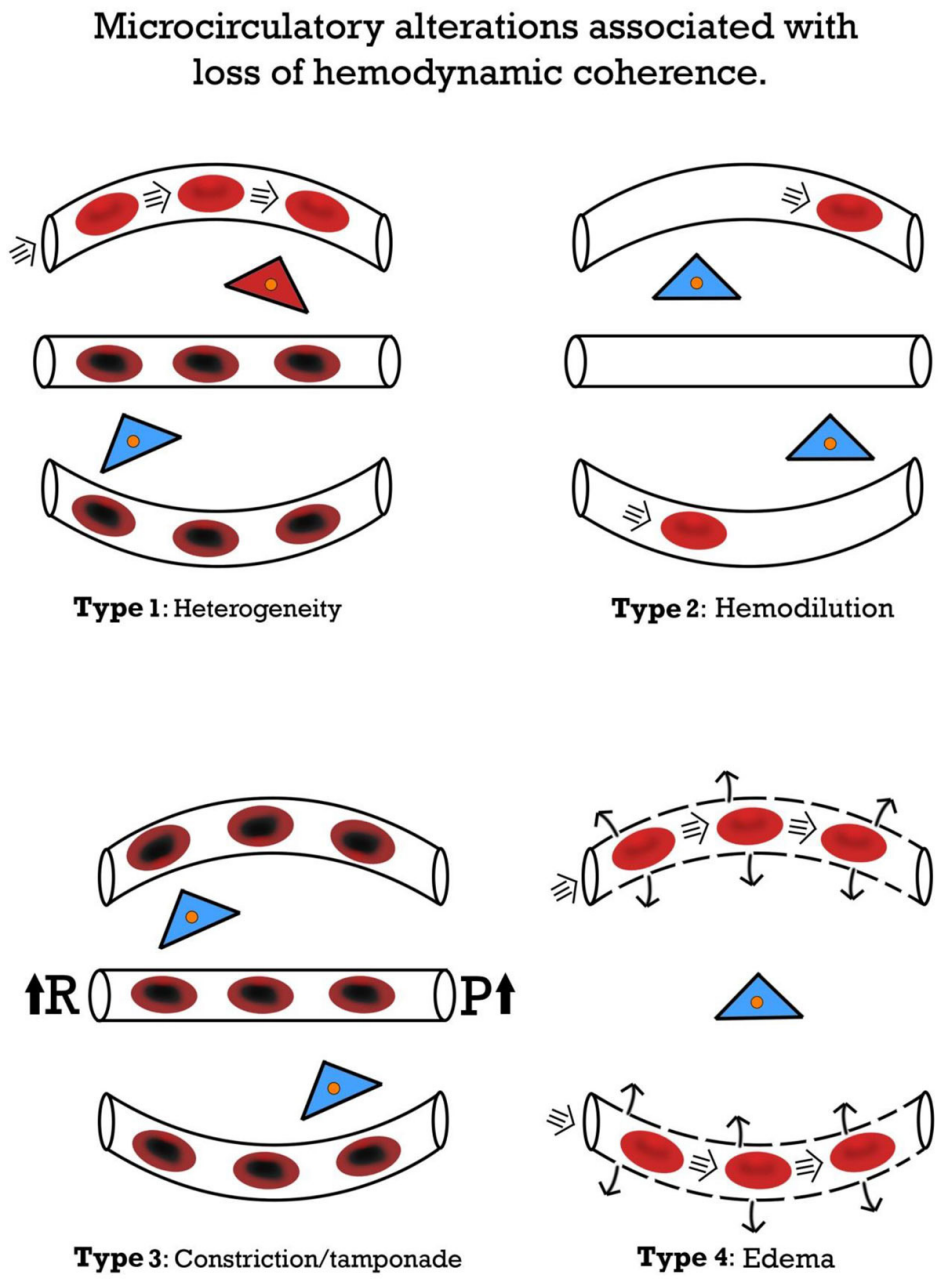
**Microcirculatory alterations associated with loss of hemodynamic coherence**. Microcirculatory alterations underlying the loss of hemodynamic coherence between the macrocirculation and the microcirculation resulting in tissue hypoxiaType 1: heterogeneous perfusion of the microcirculation as seen in septic patients with obstructed capillaries next to perfused capillaries resulting in a heterogeneous oxygenation of the tissue cells. Type 2: hemodilution with the dilution of microcirculatory blood resulting in the loss of RBC-filled capillaries and increasing diffusion distance between RBCs in the capillaries and the tissue cells. Type 3: stasis of microcirculatory RBC flow induced by alterd systemic variables (e.g. increased arterial vascular resistance(R) and or increased venous pressures causing tamponade 4 alterations involve edema caused by capillary leak syndrome and which results in increased diffusive distance and reduced ability of the oxygen to reach the tissue cells. *Red*, well-oxygenated RBC and tissue cells; *purple*, RBC with reduced oxygenation; *blue*, reduced tissue cell oxygenation

Various types of microcirculatory alterations associated with different states of cardiovascular compromise have been classified based on direct observation of the microcirculation in various organ beds [36]. Each of these classes of microcirculatory alterations is associated with a reduced functional capillary density (FCD) and thereby a loss of the capacity of the microcirculation to transport oxygen to the tissues. These losses can occur individually or in combination, especially in conditions with multiple resuscitation modalities. Reduction in the FCD can promote functional shunting of oxygen transport to the tissues, which, unless explicitly monitored, cannot be detected by simply monitoring the macrocirculation [37]. Four types of microcirculatory alterations underlying the loss of hemodynamic coherence can be identified (Figure 1): type 1, heterogeneity in microcirculatory perfusion with obstructed capillaries next to capillaries with flowing red blood cells (RBCs); type 2, hemodilution, in which dilution of blood causes a loss of RBC-filled capillaries and results in increased diffusion distances between the oxygen-carrying RBCs and tissue cells; type 3, vasoconstriction/tamponade where vasoconstriction of arterial vessels results in microcirculatory ischemia or raised venous pressures inducing microcirculatory tamponade, both resulting in compromised tissue oxygenation; and type 4, tissue edema caused by capillary leak, resulting in increased diffusion distances between the RBCs and tissue cells.

Type 1 alterations typically can be observed in septic patients. The persistence of this type in the presence of normalized systemic variables has been associated with organ dysfunction and mortality. De Backer et al. [10,32] showed in several studies in septic patients that patients with such a type 1 loss of hemodynamic coherence between microcirculatory alterations and systemic hemodynamic variables were associated with adverse outcomes. Top et al. [38] reported that such alterations can also occur in pediatric patients requiring extracorporeal membrane oxygenationsupport, and that these alterations were also associated with adverse outcomes. Edul et al. [11] specifically identified the heterogeneous nature of type 1 microcirculatory alterations as a phenotype in septic patients as being associated with adverse outcome. Trzeciak et al. [12] identified type 1 alterations that persisted despite early goal-directed therapy to be associated with the development of organ dysfunction, and the authors introduced a heterogeneity index to describe such type 1 alterations. Studies have shown that type 1 alterations result from microcirculatory distributive alterations caused by the action of various cellular insults, including RBC obstructions caused by endothelial cell dysfunction, and/or hemorheological alterations, and/or loss of or abnormal vasomotor tone due to, for example, nitrosative and or oxidative stress [39]. Type 1 abnormalities signal the need for anti-inflammatory and antibacterial agents to protect the various components of the microcirculation and vasoactive medication (e.g., vasodilators) to promote patency.

Type 2 alterations, associated with hemodilution, have been described mainly in cardiac surgery patients in whom hemodilution, caused by priming solutions, cardioplegia, and fluid administration, results in a loss of RBC-filled capillaries. This results in an increase in diffusion distance between the capillaries and the tissue cells (e.g., [40]). This reduction in oxygen-carrying capacity can be considered iatrogenic and results in hospital anemia, a condition which has been found to be a risk factor for developing organ dysfunction, in particular renal failure [41,42]. Hemodilution also results in altered viscosity that leads to loss of sheer stress, which is one of the main physiological components needed for flow-dependent vasoregulation essential for establishing hemodynamic coherence [43]. In experimental studies, we found that hemodilutional anemia can cause the loss of oxygen availability in an organ-dependent manner, with the kidney being the organ most vulnerable to hemodilution [44]. Such a hemodilutional loss of coherence can be corrected by maintaining an adequate hematocrit and by the appropriate administration of quality blood (e.g., [45,46]).

Type 3 loss of coherence has been demonstrated in several clinical studies and involves the sometimes unintentional and undetected manipulation of systemic variables that leads to stasis or tamponade of microcirculatory flow. Vasopressor therapy is a standard of care in the treatment of sepsis and shock and is used to increase blood pressure, although such a maneuver can be associated with adverse outcomes [47]. Several microcirculation studies in patients have shown a loss of coherence whereby blood pressure rises but microcirculatory RBCs fall (e.g., [48]). Dubin et al. [49] and Janji et al. [50], in independent studies, showed this to be the case in septic patients treated with norepinephrine. A similar loss of coherence was found by Hernandez et al., who investigated the use of dobutamine in septic shock patients. Despite an increase in cardiac index, heart rate, and left ventricular ejection caused by the dobutamine administration, no beneficial effect on sublingual microcirculation was found as observed by handheld microscopy [51]. Hyperoxia also falls into the category of type 3 loss of hemodynmic coherence. In this type alteration, therapeutic increases in inspired oxygen fraction are expected to improve the oxygen availability in the tissues can have deleterious effects on the microcirculation and also have adverse effects on patient outcomes [52]. Cortés et al. [53] demonstrated in volunteers that inhalation of raised fractions of oxygen causes a reduction in the FCD and flow in the sublingual microcirculation. Guidelines recommend that resuscitation should be associated with an increase in venous pressure [54], although elevated venous pressures have been associated with adverse outcomes, specifically in terms of renal function [55]. From a physiological point of view, raised venous pressure may reduce microcirculatory perfusion due to tamponade. Evidence to support this etiology was demonstrated by Vellinga et al. [56], who compared patients with venous pressure higher than 12 mmHg with those with lower pressures, and found that there was a significant reduction in microcirculatory perfusion in those patients with higher venous pressures.

Type 4 microcirculatory alteration is associated with tissue edema caused by capillary leak and edema due to endothelial cell damage, loss of glycocalcyx barriers, and/or compromise of adherens and tight junctions. Tissue hypoxia in edema is fueled by increased diffusion distances between the RBC filled capillaries and tissues cells caused by an accumulation of tissue water in combination with the poor oxygen solubility and therefore transport in tissue water. Because such a condition may be associated with systemic indicators of hypovolemia, it may invite further administration of fluids, which can cause even more tissue edema and organ dysfunction. Such a condition may, in part, explain the adverse outcomes resulting from the liberal fluid administration in the Fluid Expansion as Supportive Therapy (FEAST) trial trial [57] that involved a mixed population of African children with sepsis and malaria. The PRISM (PiCCO-guided Resuscitation in Severe Malaria) trial trial in malaria patients showed that guiding fluid therapy based on the correction of systemic hemodynamic using the PICCO technique in conditions of type 4 alterations occurring in malaria patients can result in adverse outcomes. This approach resulted in marked generalized peripheral edema and did not reverse signs of hypovolemia and acidosis, whereby patients ultimately required renal replacement therapy [58]. The central role of type 4 microcirculatory alterations in the setting of malaria patients was elegantly demonstrated by Hanson et al. [59] in a rectal microcirculation study in malaria patients. They showed that stroke volume-guided administration of fluids was successful in increasing the global end-diastolic volume index but had little effect on RBC sequestration. This primary pathology in malaria was directly observed using hand-held microscopy. Fluid administration based on systemic variables was also not able to correct acid-base alterations or lactate acidosis, but instead promoted tissue edema in the lungs and kidney [59].

## Targeting the microcirculation to resolve coherence

Evaluation of the success of resuscitation based on the macrocirculation should be accompanied by a verification of the recruitment of the microcirculation. Several studies have shown that the finding of an initial low microcirculatory flow independent of the value of systemic hemodynamic variables predicts the success of microcirculatory responsiveness to resuscitation procedures such as fluid therapy, blood transfusion, and vasopressor administration [49,60,61]. Surrogates of organ perfusion, such as lactate, peripheral temperature, and capillary refill time, can be used to identify the loss of hemodynamic coherence. Alterations in these variables have been found to identify organ dysfunction [62], and these alterations have been suggested for use as resuscitation end points [63]. Although evaluation of peripheral circulation at the bedside is relatively easy, there may be some drawbacks. For example, lactate represents a downstream metabolic product whose origin may not always reflect lack of organ perfusion [64]. Many peripheral perfusion indicators, such as peripheral temperature and capillary refill, are based on the skin. However, the function of microcirculatory perfusion in the skin is primarily related to functions other than tissue oxygenation, such as thermoregulation. In a study in septic patients, a lack of coherence was found between abnormal peripheral temperature and alterations in sublingual microcirculation [65]. Edul et al. [35] also found no correlation between the finger to core temperature difference and the intestinal microcirculation, whereas abnormal intestinal microcirculation alterations correlated with increased mortality. In a nitroglycerin resuscitation study in septic patients, Boerma et al. [66] found that nitroglycerin affected skin temperature but had no effect on the sublingual microcirculation. In addition to these drawbacks, peripheral perfusion abnormalities give little information about the origin of the loss of hemodynamic coherence. Direct observation of the nature of microcirculatory alterations using hand-held microscopes, however, allows a more detailed insight into the nature of microcirculatory alterations and indicates which therapeutic strategies would best correct the type of alteration observed (Figure 1).

For the aforementioned reasons, direct visualization of flowing RBCs in the microcirculation using hand-held microcopy as method to assess the functional state of the microcirculation must be considered the gold standard for tissue perfusion. The use of hand-held vital microscopy, clinically introduced by us, has gone through a number of technological developments with the ultimate aim of introducing these devices for routine clinical use. In this way, three generations of bedside hand-held microscopes have been developed [67]. The first-generation devices were based on a combination of orthogonal polarization spectral imaging and dark-field illumination and called the Cytoscan. We introduced this technique clinically for the first-time observation of the microcirculation in internal organs during surgery (e.g., [68]). These first-generation devices were initially used to identify the clinical significance of microcirculatory alterations in sepsis [69] as well as the response to therapy (e.g., [70]). Based on the need to have battery-based devices, we developed a second-generation camera based on sidestream dark-field (SDF) illumination imaging called the Microscan [71]. In addition, we developed and introduced a software platform called AVA for offline analysis of the obtained movies of the microcirculation [72]. Scoring systems were developed to evaluate the microcirculation [73] and to assess the quality of the images obtained [74]. Next a laptop-based device similar to the SDF device was developed called the Capiscope [75]. The first-generation and second-generation devices had a number of shortcomings, however, which limited their applicability for routine clinical use, the most important of which was their inability to automatically analyze microcirculatory images [76]. This inability was mainly due to the limitations of the hardware in these earlier devices, which among other shortcomings lacked direct computer control of the imaging modality needed for automatic analysis. To address these shortcomings, a third-generation device called the Cytocam IDF device was recently introduced based on incident dark-field imaging [77,78]. This device consists of a computer-controlled high-resolution image sensor plus extra-short pulsed illumination needed for accurate measurement of RBC velocity. In addition to these features, the hand-held microscope has a specialized designed microscope lens that produces high-resolution images showing approximately 30% more capillaries than the previous-generation devices [78]. The hardware of this third-generation device meets the requirements for the development of an automated analysis system needed for direct identification of hemodynamic coherence, bedside clinical decision-making, and titrating therapy targeting the normalization of microcirculatory alterations shown in Figure 1.

Although the impact of many therapeutic modalities on the microcirculation have been investigated (e.g., [79]), the therapeutic intervention most investigated and potentially ready for implementation of microcirculatory-guided therapy involves the administration of fluids. The reason for this interest is that there is great controversy concerning the required composition of fluids, and there is a need for a physiological framework for administering an optimal amount of fluid based on a point-of-care basis taking the possibility of loss of hemodynamic coherence into account. Several studies have shown a loss of hemodynamic coherence between the microcirculation and the macrocirculation following fluid administration. In general, it seems that there is a benefit to the administration of fluids if microcirculatory flow is low, whereas no benefit of fluid therapy is observed if microcirculatory flow is normal or even high despite the presence of clinical surrogates of hypovolemia. This hypothesis was demonstrated elegantly in the study of Pranskunas which showed that patients with surrogates of the presence of organ hypoperfusion (e.g., lactate, tachycardia, hypotension, oliguria) could either have normal or reduced microcirculatory flow. However, only those patients with reduced microcirculatory flow benefitted from the administration of fluids, which caused an increase in microcirculatory flow and was associated with a reduction in the clinical surrogates of organ perfusion [61]. In contrast, patients who had normal flow despite the presence of the clinical surrogates of organ hypoperfusion did not respond to fluids with a change in microcirculatory flow and did not benefit with improvement in the clinical surrogates of organ perfusion. This effect was independent of the response of fluid administration on stroke volume.

A study by Ospina-Tasconet al. demonstrated the importance of the timing of fluid administration in the course of sepsis, and the authors found that early fluid resuscitation was effective in improving the microcirculation; however, giving fluids later in the course of sepsis was ineffective in improving microcirculatory hypoperfusion. Again, these effects were independent of the effect of fluid administration on the cardiac index [80]. In hypovolemic septic shock patients, Pottecheret al. found that initial fluid resuscitation was effective in improving cardiac output in synchrony with the microcirculation but not in synchrony with changes in arterial pressure. Subsequent fluid administration increased the cardiac output linearly but was associated with a much smaller response in the microcirculation, indicating that the administrated fluid volume had reached a plateau in advance of the macrocirculation [81]. In the study, they also found a reduction in type 1 microcirculatory alterations where the reported effects were independent of the use of saline or hydroxyethyl starch as a resuscitation fluid [81].

The composition of fluids used for treating hypovolemia and dehydration is a topic of controversy and is referred to as the "Great Fluid Debate" (e.g., [82]). This discussion has several components, all of which relate to the subject matter of the current article. The first component concerns the composition of the fluids. The excessive administration of 0.9% NaCl leading to chloride or dilutional acidosis being a potentially harmful procedure in the context of fluid therapy has been a primary focus of the debate [83,84]. A further matter for debate concerns the use of colloid solutions versus crystalloid solutions in the treatment of hypovolemia. In both respects, there is a need for a more physiological approach and a monitoring platform for the choice and titration of fluids. The physiological reason for choosing a colloid over a crystalloid solution is when there is a need for intravascular volume expansion such as can occur in hypovolemic shock. Indeed, Ananne et al. demonstrated this convincingly in the multicenter Colloids

**Figure 2:**
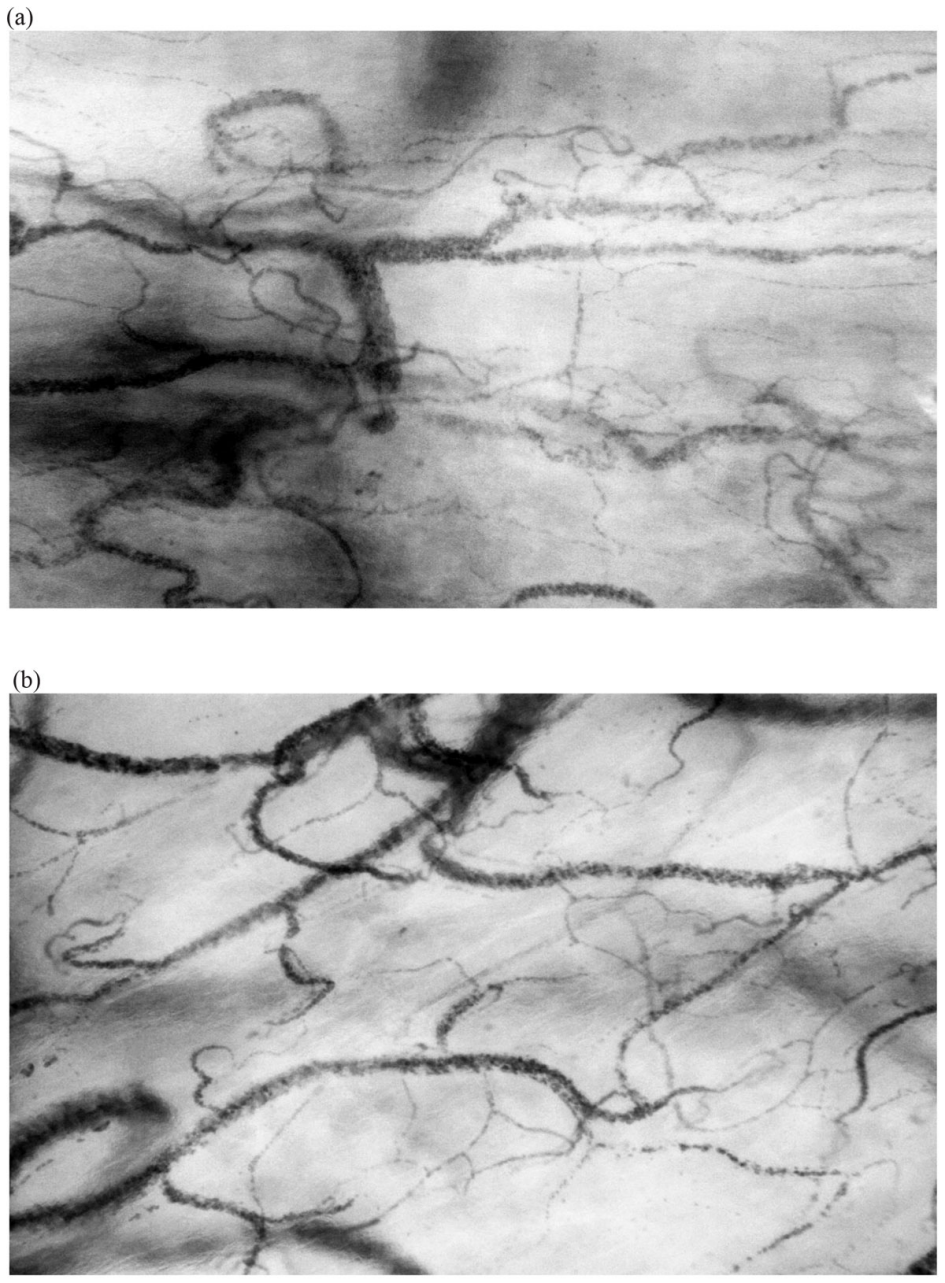
**Typical sublingual microcirculatory images taken with a Cytocam IDF hand-held microscope during cardiac surgery showing how the administration of colloid causes volume expansion while a crystalloid solution does not**. **a **Sublingual microcirculation during cardiac surgery with crystalloid 0.9% NaCl as priming solution during cardiopulmonary bypass. **b **Sublingual microcirculation during cardiac surgery with HES as priming solution during cardiopulmonary bypass. Images show that the use of HES results in more volume expansion as indicated by the increased distance between the RBCs in the capillaries as would be expected from a colloid, in comparison with **a **in which crystalloid was used in the pump where it is expected that the crystalloid solution equilibrates more rapidly with the tissues less affecting intravascular volume status.

Versus Crystalloids for the Resuscitation of the Critically Ill (CRISTAL) trial resuscitation trial involving hypovolemic patients requiring intravascular volume expansion. In comparison with the administration of crystalloids, the authors showed that colloid administration improved 90-day outcome and resulted in a higher 7-day survival without mechanical ventilation and vasopressor therapy [85]. Dubin et al. also showed that volume expansion is better achieved at the microcirculation level by the use of colloids (6% hydroxyethyl starch 130/0.4 (HES)) in septic shock patients. Not only was less HES solution needed to reach the required arterial blood pressure target than 0.9% NaCl, but HES solution was also more effective in increasing the FCD as well as the microcirculatory flow [86]. Observations using hand-held microscopy can be used to illustrate the efficacy of colloids as volume expanders. Figure 2 shows Cytocam IDF imaging of the sublingual microcirculation in cardiac surgery to compare a HES primed procedure (Figure 2b) with one primed with 0.9% NaCl (Figure 2a). The volume expansive properties of HES expansion can be observed by the increased distance between the RBCs in the capillaries in comparison with 0.9% NaCl priming (Figure 2).

Targeting tissue perfusion as a hemodynamic end point in fluid resuscitation can be considered an ultimate goal [87]. Targeting only systemic hemodynamic variables may lead to fluid overload, a potentially harmful condition. Xu et al. [88] elegantly showed that targeting the microcirculation results in much less fluid administration than does targeting systemic variables with the same outcome. In a pig hemorrhagic shock model, they withdrew 1 l of blood, followed by blood and fluid resuscitation that targeted the normalization of either the blood pressure or the sublingual microcirculation. Both resuscitation procedures were equally effective in terms of outcome and even neurological function, but the correction of blood pressure to baseline required 300 ml blood and 700 ml Ringer's lactate while targeting the microcirculation only required 174 ml Ringer's lactate with no blood being needed. This study showed that the amount of administered fluids is more dependent on the hemodynamic parameter chosen as the target than on the success of resuscitation.

**Figure 3:**
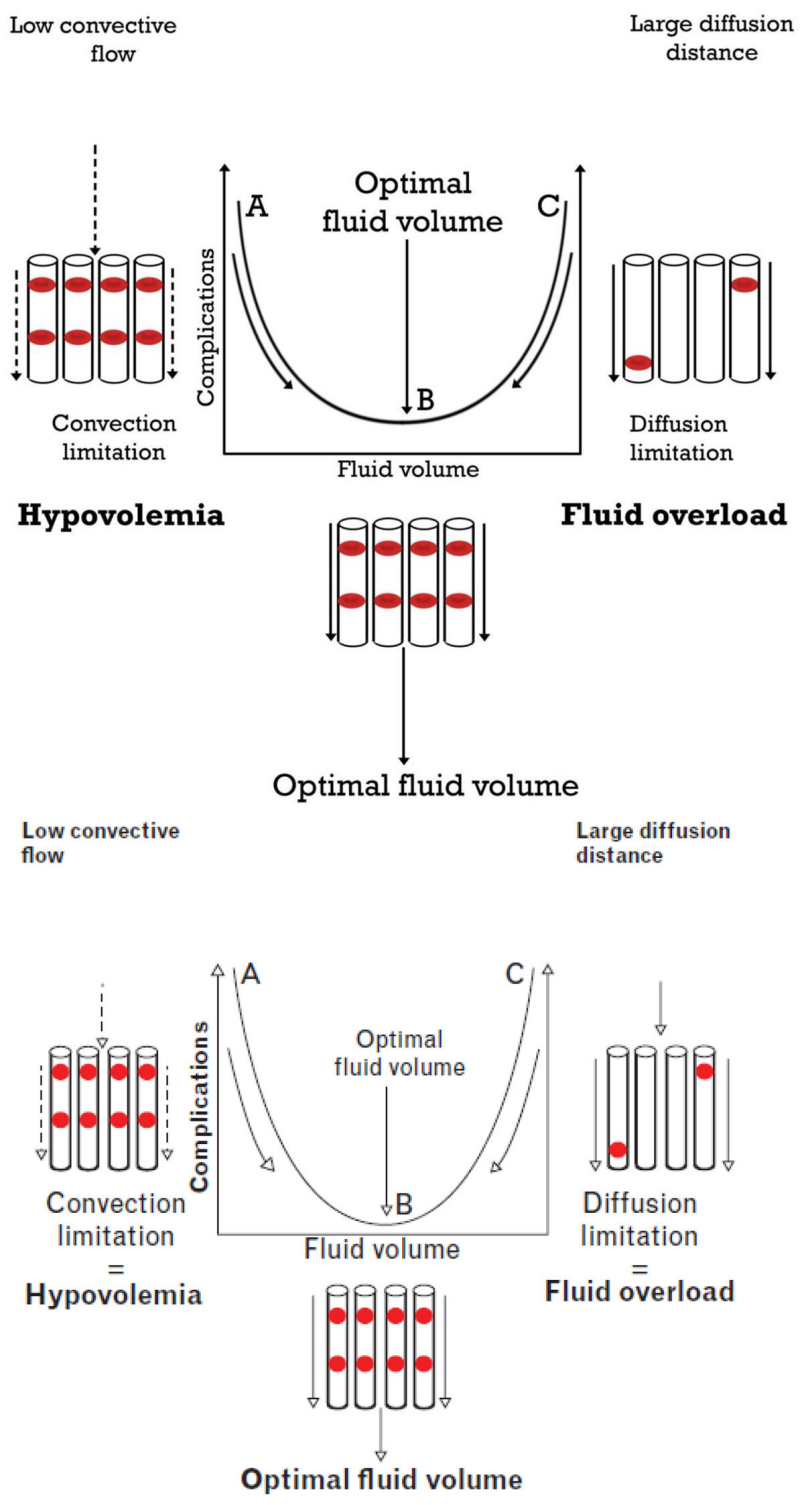
**Microcirculatory-guided fluid therapy**. To optimize the oxygen-carrying capacity of the microcirculation, optimization is required of the convective (sufficient flow) and diffusive capacity (optimal FCD to have short diffusion distances between the oxygen-carrying RBCs and the tissue cells). Observation of sublingual microcirculation using hand-held microscopy in states of hypovolemia identifies low convective flow (*left*), indicating the need for fluid administration. Microcirculatory fluid responsiveness indicates the success of fluid therapy by showing enhanced convective RBC flow. A reduction in FCD signals the occurrence of a type 2 microcirculatory alteration (*right*) and this indicates that too much fluid has been administered, causing increased diffusion distance between the RBCs and tissue cells reducing the oxygen transport capacity of the microcirculation. This approach provides a personalized physiological-based patient-centered fluid resuscitation strategy to optimize the oxygen-carrying capacity of the microcirculation. Adapted from [89].

Based on the aforementioned background, we proposed a physiological rationale for targeting the microcirculation to administer optimal fluid volumes in a personalized setting [Figure 3] [89]. In this view, hypovolemia is defined by low microcirculatory convective flow resulting in poor oxygen transport and in the need for fluid administration. The success of fluid administration is identified as an improvement in convective flow, and this improvement defines the success of fluid administration [61]. In conditions of low flow which defiens and identifies the presence of hypovolemia (left side of Figure 3), fluids should be administered until there are optimal microcirculatory flowing RBCs filling the capillaries (center of Figure 3). However, too much fluid will induce excessive dilution and a type 2 alteration will follow whereby there will be an insufficient number of RBCs to fill capillaries, resulting in a reduction in the diffusive capacity of oxygen transport to the tissues in the microcirculation (right side of Figure 3). This condition indicates a need to stop giving fluids to avoid fluid overload identifying the optimal microcirculatory response to fluid therapy. It is proposed that this model, based on optimizing the oxygen-carrying capacity of the microcirculation using hand-held microscopy, may provide a physiological basis for administering an optimal volume of fluid to the individual patient [89].

## Conclusion

Clearly the ultimate aim of resuscitation is the restoration of perfusion of vital organs and tissues where oxygen supply to the tissues is compromised due to shock [63]. To accomplish this end point, oxygen-carrying RBCs must successfully enter the microcirculation and deliver oxygen to the tissues. Conventional resuscitation procedures including fluids, vasoactive medication, and blood are clinically administered to accomplish this aim. However, the physiological variables used to target resuscitation are based on correcting systemic hemodynamic variables of pressure, flow, and/or oxygen delivery. Knowing whether these procedures are successful in achieving adequate perfusion and oxygen transport to the organ tissues is unknown at the bedside, and relies on the assumption that there is hemodynamic coherence between the macrocirculation and the microcirculation. With the introduction of hand-held microscopy at the bedside, the nature of microcirculatory alterations has been elucidated. These alterations have been found to have clinical significance in terms of morbidity and mortality, and hand-held microscopy can identify microcirculatory alterations that underlie the loss of hemodynamic coherence. In this article, we identified four types of such microcirculatory alterations underlying the loss of hemodynamic coherence. Studies have shown that microcirculatory alterations are associated with adverse outcomes in a manner that seems to be independent of systemic hemodynamic variables. Fluid therapy can be effective in recruiting the microcirculation in states of hypovolemia, but the choice of composition and volume remains a source of debate. In this article we propose a model for optimizing volume administration based on the microcirculation. The introduction of a new generation of hand-held microscopy [78] with the potential of automatic analysis opens the way to enabling titration of fluids to optimally recruit the microcirculation in such a way as to optimize its oxygen transport capacity. Future clinical trials will be needed to determine whether such procedures will translate into improved outcomes in comparison with fluid therapy based on systemic hemodynamic variables.

## Abbreviations

FCD, Functional capillary density; HES, 6% Hydroxyethyl starch 130/0.4; RBC, Red blood cell; RCT, Randomized controlled trial; SDF, Sidestream dark field.

## Competing interests

In the last period of 2 years CI has received honoraria and independent research grants from Fresenius-Kabi (Bad Homburg, Germany), Baxter Health Care (Deerfield, IL, USA), and AM-Pharma (Bunnik, the Netherlands). CI has developed SDF imaging and is listed as inventor on related patents commercialized by MicroVision Medical (MVM, Amsterdam, The Netherlands) under a license from the Academic Medical Center, Amsterdam, The Netherlands. He has been a consultant for MVM in the past, but has not been involved with this company for more than 5 years now, except that he still holds shares. Braedius Medical, a company owned by a relative of CI, has developed and designed the hand-held microscope called Cytocam IDF. CI has no financial relation with Braedius Medical of any sort; that is, never owned shares, or received consultancy or speaker fees from Braedius Medical.
